# Gabapentin in Burns

**DOI:** 10.5812/atr.6471

**Published:** 2014-03-15

**Authors:** Nicholas Sheppard

**Affiliations:** 1 Department of Plastic Surgery, Norfolk and Norwich University Hospital, Colney Lane, Norwich, UK

**Keywords:** Burns, Pruritus, Gabapentin

Dear Editor,

I read with interest the article entitled Effect of Gabapentin on Morphine Consumption and Pain after Surgical Debridement of Burn Wounds: A Double-Blind Randomized Clinical Trial Study and was impressed by the scientific rigor and level of detailed observation. This is especially admirable in a study performed at the front line of burn surgery. Gabapentin appears to have a role to play in the reduction of pain and opiate usage during major burns surgery. It is worth, however, bringing the readers’ attention to another key role for the drug in burn which is not mentioned in the discussion. Gabapentin also has an important role in the management of burn-induced itch. Evidence is emerging that it is more effective than conventional treatments such as antihistamines and should be considered a first-line treatment in burn patients (1). Taking this research on gabapentin in perioperative pain to the bedside and introducing gabapentin early in the treatment process for analgesia may help to prevent the transition from pain to itch that so frequently occurs during the healing process in these patients.
